# When the Healthcare System Breaks a Heart: A Case of Stress Cardiomyopathy Following a Conversation With an Insurance Provider

**DOI:** 10.1155/cric/9942574

**Published:** 2026-02-19

**Authors:** Kramer J. Wahlberg, Friederike K. Keating, Salmaan Mumtaz, Krina Patel, Ahmed A. Harhash

**Affiliations:** ^1^ Department of Medicine, Division of Cardiology, The Robert Larner MD College of Medicine, University of Vermont, Burlington, Vermont, USA, uvm.edu

**Keywords:** case report, stress, stress cardiomyopathy, takotsubo

## Abstract

Stress related to healthcare has been associated with adverse outcomes for patients with chronic conditions, including cardiovascular disease. We describe the case of an otherwise healthy patient who developed stress cardiomyopathy triggered by an emotional conversation with their healthcare insurance provider. A conversation regarding coverage that included preventative care for an asymptomatic patient resulted in the development of a cardiovascular emergency, transfer to a tertiary care cardiology service, antithrombotic therapy, and invasive procedures, all of which carried inherent risk and significant cost. The case illustrates the presentation, workup, management, and natural history of typical stress cardiomyopathy while providing the opportunity to highlight the brain–heart axis and consequences of healthcare‐related stress.

## 1. Introduction

Stress cardiomyopathy, also known as takotsubo cardiomyopathy or “broken heart syndrome,” is characterized by acute myocardial injury and transient left ventricular (LV) dysfunction in the absence of obstructive coronary artery disease (CAD) classically preceded by a stressful trigger [1]. The pathophysiology of stress cardiomyopathy is not fully understood, with proposed mechanisms involving the brain–heart axis including acute myocardial stunning secondary to catecholamine and neuropeptide‐mediated microvascular dysfunction following a stressor [2]. Extensive case series have described a variety of emotional and physical triggers; however, stress cardiomyopathy following a conversation with a healthcare insurance provider has not been described. Skyrocketing healthcare costs in the United States are accompanied by an increasing burden of healthcare‐related stress and financial hardship, which may ultimately have negative consequences for cardiovascular outcomes [3, 4] and population health [5]. The aim of this case report is to highlight the prevalence and implications of healthcare‐related stress in the United States through the lens of a novel stress cardiomyopathy trigger.

## 2. Case Presentation

A 63‐year‐old female with no history of cardiovascular disease developed an acute sensation of “feeling something was wrong” immediately following a stressful conversation with her insurance provider during which she experienced strong feelings of frustration and anger. The phone conversation was regarding a change in health insurance plans. Due to persistent symptoms, presyncope, and an erratic heart rate by home pulse oximetry, the patient presented to a local emergency department where they were found to have an ECG demonstrating transient anterolateral ST elevations (Figure 1) and elevated high‐sensitivity troponin I (248 ng/L). Symptoms and ST elevations spontaneously improved without treatment. The treating physicians at the referring hospital elected to treat with aspirin, clopidogrel, and heparin infusion, and she was transferred to the cardiovascular care unit at our tertiary care academic medical center for further evaluation and management of high‐risk non‐ST elevation myocardial infarction (NSTEMI).

Figure 1Select ECGs throughout the clinical course demonstrating the typical progression of ECG abnormalities in stress cardiomyopathy. (a) Initial ECG following acute onset of symptoms, showing anterolateral and high‐lateral ST elevations. (b) Several hours after initial presentation, ECG with interval resolution of ST elevations. (c) 48 h after initial presentation, ECG evolved into dynamic, deep T‐wave inversions with prolongation of QTc interval. (d) ECG prior to discharge demonstrating improving ST and T‐wave abnormalities with interval resolution of prolonged QTc‐interval.

**Figure:**
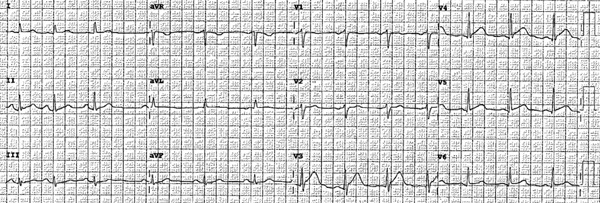
(a)

**Figure:**
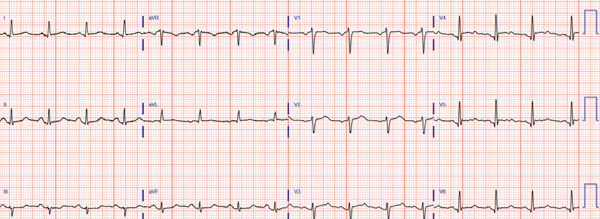
(b)

**Figure:**
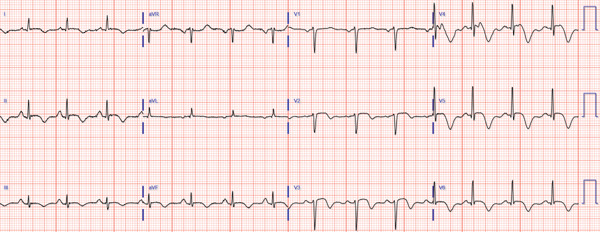
(c)

**Figure:**
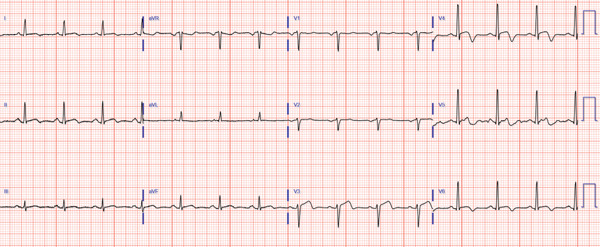
(d)

On initial examination she was found to have sinus tachycardia with normal blood pressure and was oxygenating well on room air. There were warm extremities, normal heart sounds without murmur, clear lungs, and absence of jugular venous distention or edema. There were no arrhythmias on telemetry monitoring. Troponin I dynamically increased to 4900 ng/L over several hours. Other labs included elevated NT‐proBNP (665 pg/mL) with unremarkable metabolic, blood count, and coagulation profiles.

At baseline, the patient was functionally independent and exercised without limiting symptoms. Medical history included breast cancer with historically normal baseline LV function from transthoracic echocardiograms (TTE) obtained during surveillance of cancer therapy.

Transthoracic echocardiogram obtained during current admission demonstrated LV systolic dysfunction with ejection fraction (EF) 35%, akinesis of all mid‐apical LV segments with hyperkinetic basal segments, and no LV thrombus (Figure 2). Stress cardiomyopathy was suspected given the pattern of LV apical ballooning and wall‐motion abnormalities (WMA) that involved more than one vascular territory. Left heart catheterization with coronary angiography was performed that demonstrated no angiographically significant CAD, ruling out a plaque rupture causing Type 1 myocardial infarction (Figure 3). During the first 48 h of admission, serial ECGs demonstrated dynamic, diffuse deep T‐wave inversions and corrected QT‐interval (QTc) prolongation (Figure 1).

Figure 2Apical 4‐chamber (a) and 2‐chamber frames (b) at end‐systole from admission contrast‐enhanced transthoracic echocardiogram demonstrating a dilated left ventricle with “ballooning” of the apex and akinesis of the mid and apical segments (yellow arrows). Images (c) and (d) are frames from the follow‐up echocardiogram obtained several weeks after discharge demonstrating interval recovery of the stress cardiomyopathy. The left ventricular apex is no longer dilated, and the white arrows indicate normal contraction of the mid and apical segments.

**Figure:**
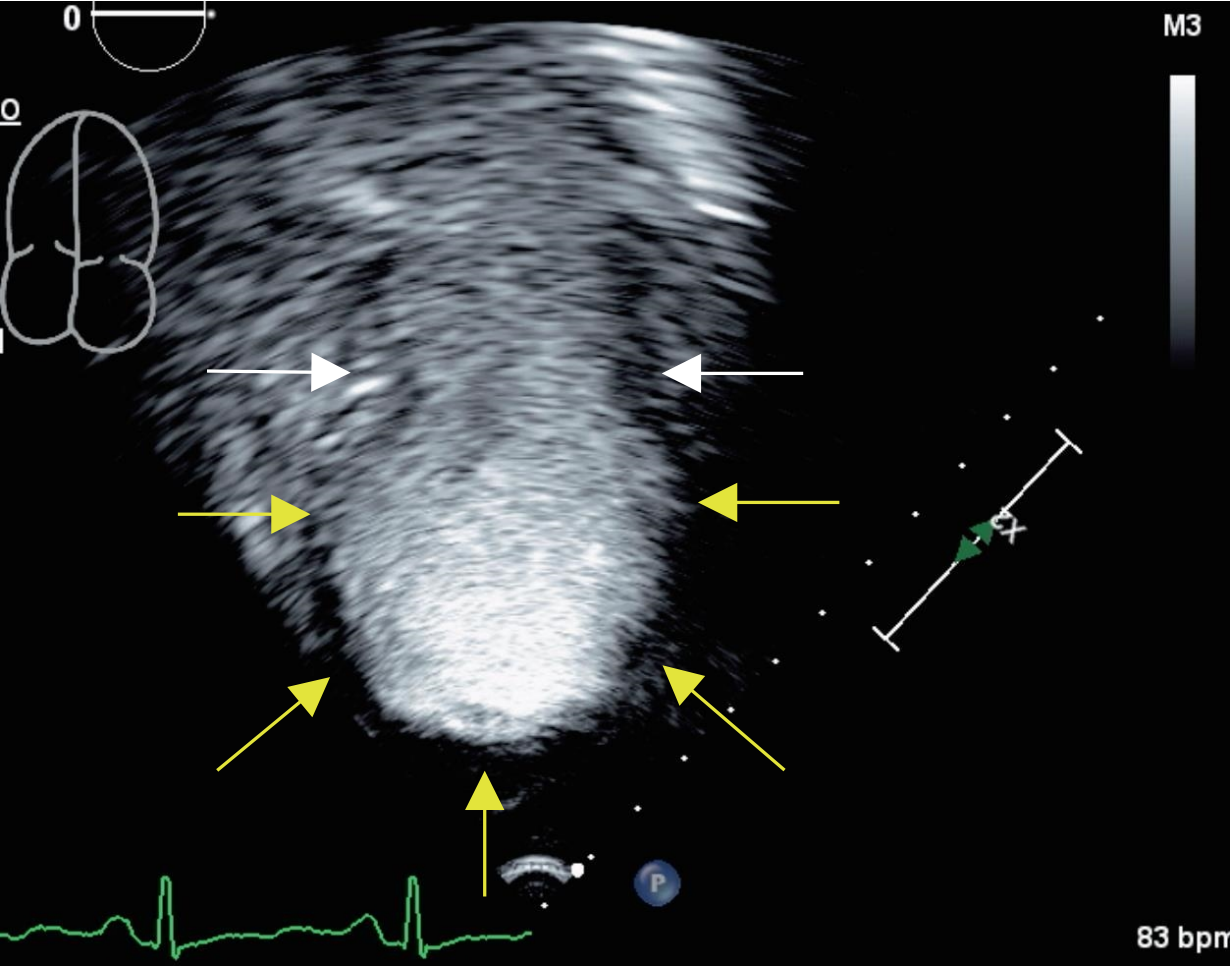
(a)

**Figure:**
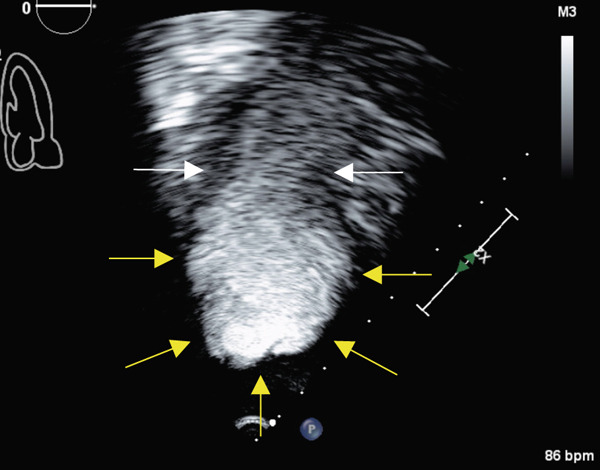
(b)

**Figure:**
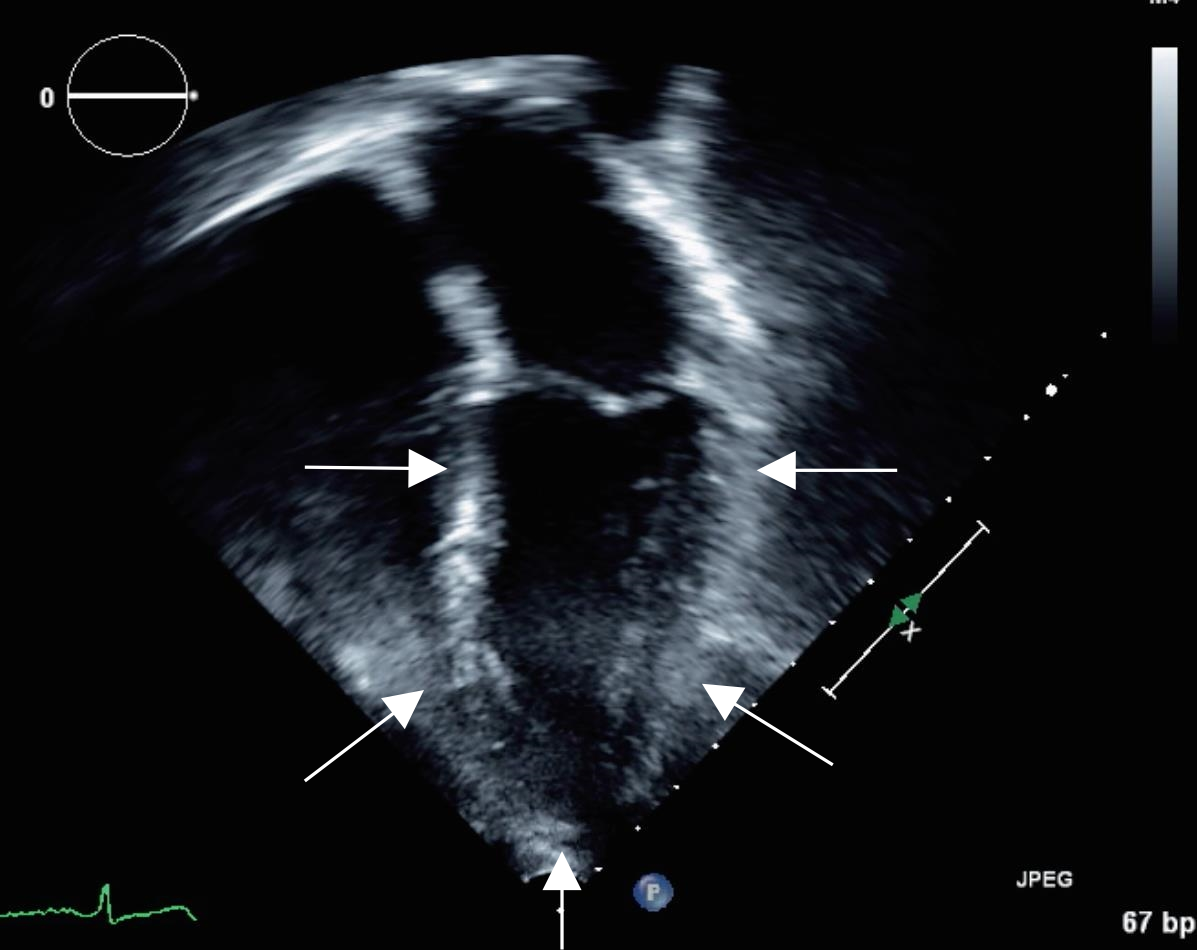
(c)

**Figure:**
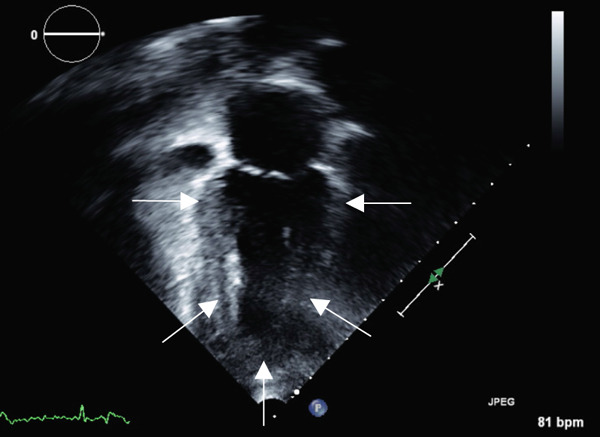
(d)

Figure 3Angiograms of the right (a) and left (b) coronary arteries demonstrating no angiographically significant coronary artery disease.

**Figure:**
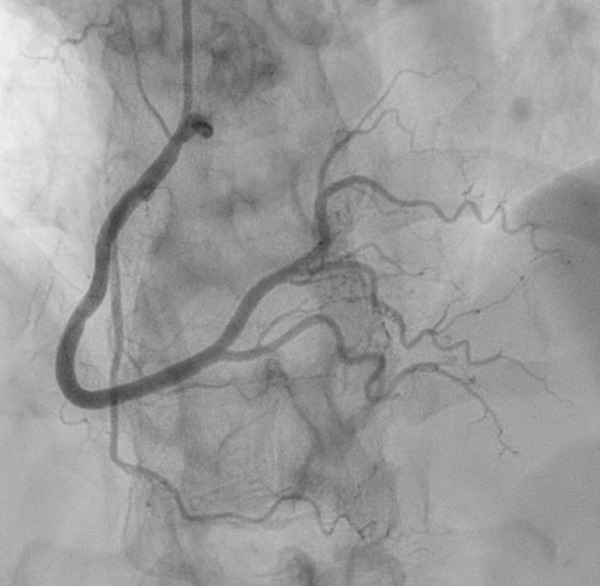
(a)

**Figure:**
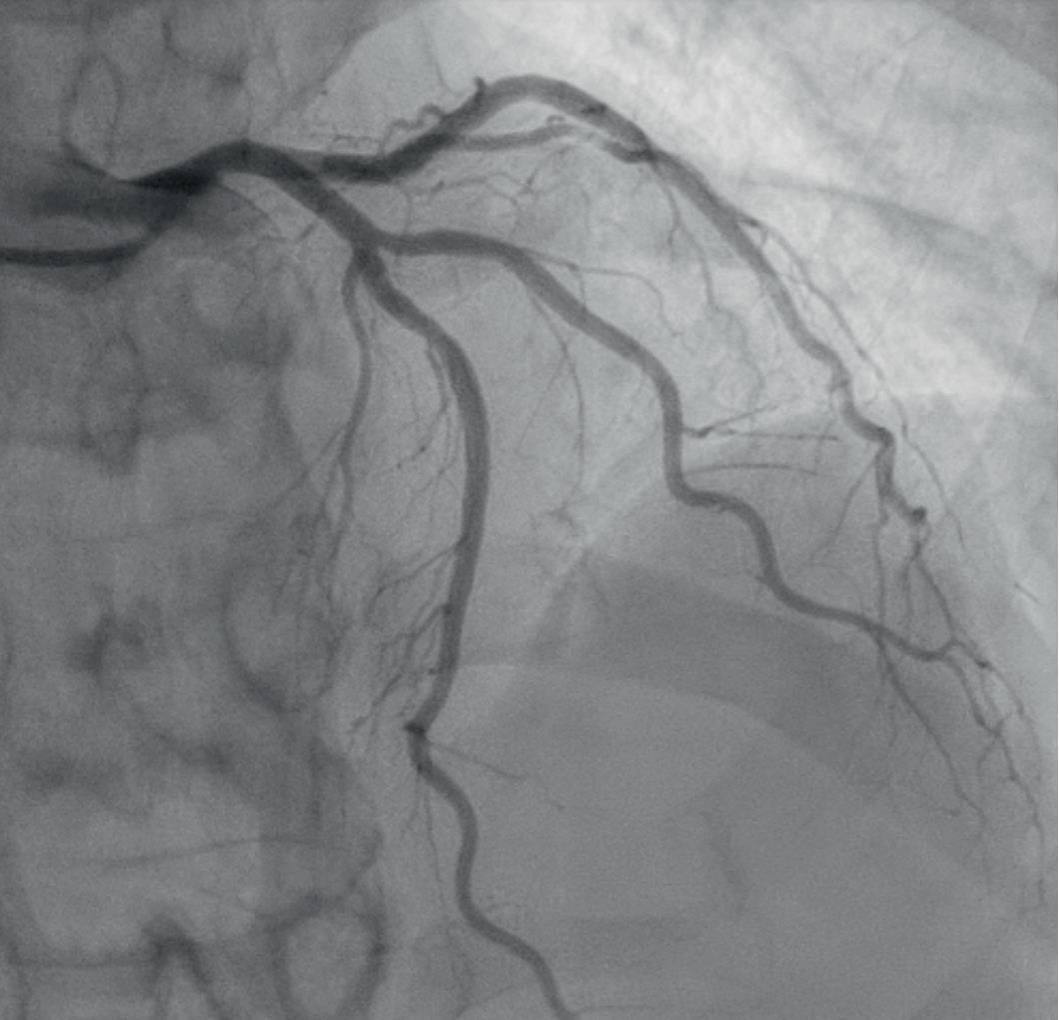
(b)

Following symptom resolution, hemodynamic stability, and improvement of ECG abnormalities, the patient was discharged to home with a diagnosis of stress cardiomyopathy. Discharge medications included low‐dose aspirin 81 mg daily, atorvastatin 40 mg daily, and metoprolol succinate 50 mg daily. Upon close outpatient cardiology follow‐up 3 weeks later, functional status had returned to baseline. TTE demonstrated recovery of LVEF and resolution of WMA. At this follow‐up visit, atorvastatin and aspirin were discontinued as there was no primary indication for prevention given suspected stress cardiomyopathy and normal coronary arteries. Similarly, the beta blocker was weaned given recovery of LVEF and lack of any established benefit of beta blockers in preventing recurrent stress cardiomyopathy.

## 3. Discussion

Stress cardiomyopathy has become increasingly recognized in clinical practice since it was first described in the 1990s–2000s [6] and, although LV dysfunction is typically reversible, it confers a high risk of mortality approaching that of acute coronary syndromes (ACS). This case describes a classic case of “typical” stress cardiomyopathy following emotional stress. Although this particular emotional trigger of stress cardiomyopathy has not been previously described, our aim with this report was to review the brain–heart axis and highlight the prevalence of healthcare‐related stress and the consequences for health.

In this case, a healthy woman′s phone call with her healthcare insurer regarding coverage for services resulted in emotional stress precipitating stress cardiomyopathy, a serious condition carrying both short‐term and long‐term risks. The acute presentation can be complicated by heart failure, cardiogenic shock, arrhythmias, and thromboembolism, with an observed mortality similar to patients with traditional plaque rupture ACS [7]. In some cases, marked QTc‐prolongation from stress cardiomyopathy can precipitate torsades de pointes and may warrant discontinuation of QT‐prolonging and heart rate lowering medications. Patients with recovered stress cardiomyopathy are at risk of recurrence observed at 2%–4% per year, a rate much higher than the risk of stress cardiomyopathy in the general population [2].

Fortunately, this patient experienced no major complications and has returned to baseline state of health with recovery of LVEF. Although stress cardiomyopathy may be an extreme example of the consequences of acute stress on the human body, researchers have long established the impact of acute and chronic stress on overall health [5] and cardiovascular disease [3] with current American Heart Association and American College of Cardiology guidelines recommending routine screening for psychosocial stressors for the primary prevention of atherosclerotic cardiovascular disease [8]. Mechanisms of detrimental stress‐mediated effects include upregulation of the hypothalamic–pituitary‐adrenocortical axis and the sympathetic nervous system, the latter thought to be a key component of stress cardiomyopathy pathophysiology [2]. Although the specific pathophysiology of stress cardiomyopathy remains uncertain, emotional triggers are common, and most patients suffering from stress cardiomyopathy suffer from a concurrent psychiatric or neurological condition which implicates the brain–heart axis. Purported mechanisms include myocardial damage and toxicity from circulating neuropeptides, catecholamines, and cortisol, as well as activation of neurons in the brain that directly innervate the coronary microcirculation leading to myocardial stunning [2, 7, 9]. Histopathological studies have demonstrated regional differences in *β*_2_ receptors and sympathetic nerve fiber distribution between the basal and apical myocardium, signaling a role for increased sympathetic activity and circulating catecholamines as a mechanism by which emotional stress could trigger the regional myocardial dysfunction characteristic of stress cardiomyopathy [9, 10].

Stress is ubiquitous. Up to two‐thirds of Americans report that the cost of healthcare and healthcare insurance are critical sources of stress [11]. Unsurprisingly, healthcare‐related stress was higher in those who were uninsured, lived with chronic medical conditions, or recently dealt with changes in healthcare coverage [11, 12]. Healthcare‐related stress was the trigger of stress cardiomyopathy in this case where discussions regarding insurance coverage unfortunately led to a costly hospitalization and invasive testing. Stress cardiomyopathy triggered by healthcare‐related stress has been described during the COVID‐19 pandemic [10]. Escalating healthcare costs and the associated financial toxicity further complicate stress related to insurance coverage. Cardiovascular conditions such as atherosclerotic cardiovascular disease and heart failure are leading causes of mortality, as well as cost of care. Up to half of patients living with these chronic medical conditions and their families report significant financial hardship related to out‐of‐pocket healthcare expenses [4, 13], and low‐income families seem to be disproportionately affected [14, 15]. Patients and families suffering from financial hardship suffer more stress and may defer care, ultimately leading to worse outcomes and strain on the healthcare system [4] The healthcare system, including insurers, and society as a whole should be more aware and proactive about managing healthcare‐related stress.

This case describes a unique trigger for stress cardiomyopathy, where the inciting stressor was a phone call with an insurance company. It is unfortunate that a conversation regarding coverage that included preventative care for an asymptomatic patient resulted in the development of a cardiovascular emergency, transfer to a tertiary care cardiology service, antithrombotic therapy, and invasive procedures, all of which carried inherent risk and significant cost. Although this case illustrates a life‐threatening consequence of acute stress, it may also serve to demonstrate the adverse impact of healthcare‐related stress on health itself.

## Funding

No funding was received for this manuscript.

## Consent

Consent was obtained from the patient to publish the case report and their perspectives were incorporated into the manuscript.

## Conflicts of Interest

The authors declare no conflicts of interest.

## Data Availability

Data sharing is not applicable to this article as no datasets were generated or analyzed during the current study.
